# Home-based tDCS for apathy in Alzheimer’s disease: a protocol for a randomized double-blinded controlled pilot study

**DOI:** 10.1186/s40814-023-01310-5

**Published:** 2023-05-05

**Authors:** Antonio L. Teixeira, Laís Bhering Martins, Thiago Macedo e Cordeiro, Lijin Jose, Robert Suchting, Holly M. Holmes, Ron Acierno, Hyochol Ahn

**Affiliations:** 1 Department of Psychiatry and Behavioral Sciences, McGovern Medical School, The University of Texas Health Science Center, 1941 East Road, Houston, TX 77054 USA; 2 Department of Internal Medicine, McGovern Medical School, The University of Texas Health Science Center, Houston, TX USA; 3 College of Nursing, Florida State University, Tallahassee, FL USA

**Keywords:** Alzheimer’s disease, Transcranial direct current stimulation, Neuromodulation, Apathy

## Abstract

**Background:**

Apathy is among the most common behavioral symptoms in dementia and is consistently associated with negative outcomes in Alzheimer’s disease (AD). Despite its prevalence and clinical relevance, available pharmacological and non-pharmacological strategies to treat apathy in AD have been marked, respectively, by potentially severe side effects and/or limited efficacy. Transcranial direct current stimulation (tDCS) is a relatively novel non-pharmacological method of neuromodulation with promising results. Compared to previous tDCS formats, recent technological advances have increased the portability of tDCS, which creates the potential for caregiver-administered, home use. Our study aims to evaluate the feasibility, safety, and efficacy of home-based tDCS for the treatment of apathy in AD.

**Methods/design:**

This is an experimenter- and participant-blinded, randomized, sham-controlled, parallel-group (1:1 for two groups) pilot clinical trial, involving 40 subjects with AD. After a brief training, caregivers will administer tDCS for participants at home under remote televideo supervision by research staff to ensure the use of proper technique. Participants will be assessed at baseline, during treatment (week 2, week 4, and week 6), and 6 weeks post-treatment. Dependent measures will cover cognitive performance, apathy, and other behavioral symptoms. Data about side effects and acceptability will also be collected.

**Discussion:**

Our study will address apathy, an overlooked clinical problem in AD. Our findings will advance the field of non-pharmacological strategies for neuropsychiatric symptoms, presenting a great potential for clinical translation.

**Trial registration:**

ClinicalTrials.gov, NCT04855643.

## Background

Alzheimer’s disease (AD) is the main cause of dementia and one of the great challenges of the twenty-first century [1]. An estimated 40 million people, mostly adults older than 60 years, have dementia worldwide, and this number is expected to increase significantly in the next decades. Despite ongoing advances in the understanding of AD pathogenesis, no available treatment effectively prevents or delays either the cognitive decline or the neuropsychiatric symptoms (NPS) that characterize the condition [1]. Nearly all patients with AD present with NPS, also called behavioral and psychological symptoms of dementia. NPS have been associated with negative outcomes in AD, including decrease of patient and caregiver quality of life, increased risk of institutionalization, higher costs, and risk of mortality [2]. The expression “NPS” is an umbrella expression that encompasses different types of behavioral problems, such as agitation, apathy, dysphoria, and psychosis, among others [3, 4]. Due to the potential complications associated with psychotropic drugs (e.g., increased risk of cerebrovascular events with antipsychotics, increased risk of falls, and cognitive decline with benzodiazepines) and the limited evidence of their efficacy, clinical guidelines, medical organizations, and expert groups recommend non-pharmacological strategies as the first-line treatment for NPS [5, 6].

Apathy, which is defined as the loss or reduction of interest and goal-directed behaviors, is the most common NPS in AD, with a 5-year prevalence of over 70% in this population [7, 8]. Apathy has been associated with caregiver burden, risk of institutionalization, increased costs, and greater functional impairment [9, 10]. Because of its prevalence and clinical relevance, apathy is an important target when managing AD. Standard pharmacological approaches for apathy in AD rely on cholinesterase inhibitors, such as donepezil and rivastigmine, with little evidence of therapeutic effect [10, 11]. While the stimulant methylphenidate displays some effectiveness [12], its use has been associated with increased anxiety and weight loss [13]. Another concern with stimulants is their potential cardiovascular effects, a fact particularly relevant to older adults with multiple medical comorbidities [14]. Studies investigating non-pharmacological strategies for apathy in AD, such as music, art therapy, psychomotor activity, and acupuncture, have shown modest effects in patients in the early stages of dementia [15, 16]. Therefore, there is a great need to develop effective and safe strategies for the treatment of apathy in AD.

Transcranial direct current stimulation (tDCS) is a relatively novel non-pharmacological method of neuromodulation showing promising results with depression and negative symptoms (including apathy) of schizophrenia [17, 18]. tDCS modulates brain activity through low-intensity electrical currents applied over the scalp and appears to affect network connectivity involving the prefrontal cortex and the cingulate cortex, regions implicated in the neural basis of apathy [19–21]. There is an emerging literature on the application of tDCS in AD, especially focusing on its potential cognitive effects [22–26]. For example, Smirni et al. observed improvement in performance on verbal fluency test in patients with mild AD after a 20-min session of a constant current of 1 mA with the cathode applied to the right dorsolateral prefrontal cortex [27]. Khedr et al. observed cognitive improvement as assessed by general cognition measures (i.e., Mini-Mental Status Exam and Montreal Cognitive Assessment) in patients with mild to moderate AD submitted to 2 mA anodal tDCS for 20 min on each left and right temporal lobes [28]. The results are promising despite a marked heterogeneity of the stimulation protocols (e.g., intensity, frequency, brain target).

The effect of tDCS on NPS in AD has been much less studied [23]. A previous study investigated the effect of tDCS for apathy in patients with AD, showing that the strategy was safe, but without therapeutic benefit [29]. The lack of efficacy was attributed to the short period of intervention and low number of sessions (six sessions during 2 weeks), partly because the patients needed to be taken to the medical center for stimulation [29]. Compared to previous tDCS formats, recent technological advances have increased the portability of tDCS, which creates the potential for caregiver-administered home use [30, 31]. This is an important advantage because patients with AD usually cannot drive safely, and caregivers and/or family members need to be available to bring them into tDCS sessions, which routinely require administration over several consecutive days. Given the clinical relevance of apathy in AD and the potential therapeutic effects of tDCS on this symptom, our study aims to test, as primary outcomes, feasibility, acceptability, and safety of home-based tDCS for the treatment of apathy in AD. The study will also investigate the effect of tDCS on AD-related symptoms, especially apathy, as secondary outcomes.

## Methods

### Design

This study is a randomized double-blinded controlled trial to assess the feasibility, acceptability, and safety of providing home-based, caregiver-delivered tDCS to AD patients with apathy by comparing active tDCS with sham tDCS. This study was approved by the Institutional Review Board of The University of Texas Health Science Center at Houston (UT Health Houston) (HSC-MS-21-0089) and was registered on the ClinicalTrials.gov platform (NCT04855643). This trial is funded by the Texas Alzheimer’s Research and Care Consortium (TARCC), a collaborative research effort established and funded by the State of Texas (http://www.txalzresearch.org/).

### Participant selection

**Fig. 1:**
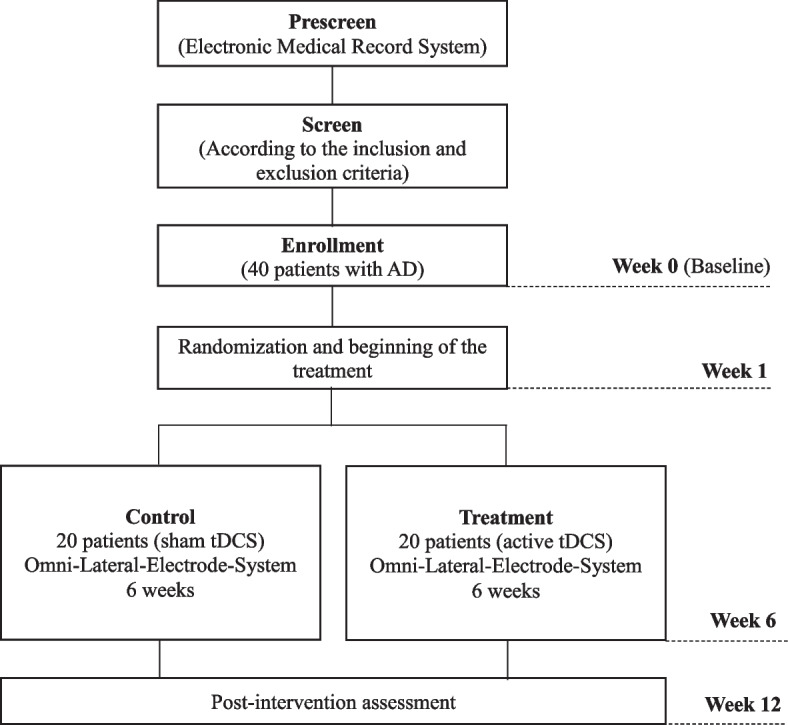
Overview of the study design. AD, Alzheimer’s disease

Participants will be recruited at UT Geriatric Neuropsychiatry Clinic, the Harris County Psychiatric Center, and the UT Physicians Center for Healthy Aging. Participants will be considered for the study if aged 60 or older and if they have the clinical diagnoses of AD and apathy. After a medical chart-based prescreening, potential candidates will be screened by the research team (Fig. 1).

Participants will be included in the study if they fulfill the following criteria (Table 1): (1) diagnosis of possible or probable AD according to the National Institute of Aging – Alzheimer’s Association diagnostic criteria [32]; (2) mild or moderate dementia, as defined by the Mini-Mental Status Exam (MMSE) score between 14 and 26 [33]; (3) clinically meaningful apathy for at least 4 weeks, as defined according to the 2018 Apathy Diagnostic Criteria [34] and/or a Neuropsychiatric Inventory (NPI-Q) apathy score equal or above 4 (i.e., severity of “moderate” or greater, caregiver distress “milder” or greater) [35–37]; (4) stable non-pharmacological approaches and doses of cholinesterase inhibitors, memantine, and other psychotropic medications for at least 2 months. The exclusion criteria include (1) unstable medical conditions, (2) history of epilepsy, (3) metallic objects in the brain, and (4) a score higher than 18 on the Cornell Scale for Depression in Dementia following an interview with the participant and the caregiver/informant [38].

**Table 1:** Inclusion and exclusion criteria

Inclusion criteria	Exclusion criteria
a) Diagnosis of possible or probable AD according to the National Institute of Aging – Alzheimer’s Association diagnostic criteria [32] b) Mild or moderate dementia, as defined by the MMSE score between 14 and 26 [33] c) Clinically meaningful apathy for at least 4 weeks, as defined according to the 2018 Apathy Diagnostic Criteria [34] or NPI-Q apathy score equal or above 4 (i.e., severity of “moderate” or greater, caregiver distress “milder” or greater) [35–37] d) Stable non-pharmacological approaches and doses of cholinesterase inhibitors, memantine, and other psychotropic medications for at least 2 months	a) Unstable medical conditions b) History of epilepsy c) Metallic objects in the brain d) Diagnosis of major depression and/or a score higher than 18 on the Cornell Scale for Depression in Dementia [38]

*Abbreviations: AD* Alzheimer’s disease, *MMSE* Mini-Mental Status Exam, *NPI-Q* Neuropsychiatric Inventory

We adopted clinical and operational criteria of apathy, the latter based on the NPI, as previously used in clinical trials for apathy [13]. The effect of tDCS on apathy will be assessed by two other tools—the Apathy Evaluation Scale (AES) and the brief Dimensional Apathy Scale (bDAS)—which provide continuous values and a more granular perspective of the syndrome. AES is the best-validated scale for measuring apathy in AD, which consists of 18 items phrased as questions that are to be scored by the clinician after the assessment of the participant and interviewing the caregiver [39]. bDAS is a short (9 items) tool based on caregiver/informant report and is specifically designed to capture different dimensions of apathy [40].

Given that lack of motivation can be a feature of depressive disorders, and apathy and depression are frequently comorbid, we expect to minimize this overlap by excluding patients with the diagnosis of major depression, also controlling for subclinical depressive symptoms through the Cornell Scale for Depression in Dementia [38].

### Randomization

Participants will be randomly allocated to either active tDCS or sham tDCS groups. Code letters (e.g., “B” and “M”) will be randomly selected and assigned to represent each treatment by the study coordinator, who will securely record and save the code assignments for eventual unblinding. The study statistician will code the randomization sequence using the random allocation rule via the *rarPar* function of the *randomizeR* [41] package in the R Statistical Computing Environment [42] to generate the entire blinded treatment allocation sequence. An unblinded collaborator will execute the randomization sequence code, record the generated allocation sequence onto a series of index cards, and place the cards into sequentially numbered opaque envelopes. Each envelope will then be opened by the unblinded study coordinator as needed throughout the trial to determine the assignment for each participant.

### Sample size

The sample size of 40 was set to maximize the number of participants that may be enrolled over the time period of the study assuming a credible average recruitment rate. As noted, the Bayesian analyses will provide the primary inferential results, as these analyses allow probabilistic interpretation of a range of effect sizes irrespective of concerns related to statistical power, and FDA guidelines have advocated for the use of Bayesian inference to improve estimates when sample sizes are small [43]. Frequentist power calculations via G*Power v. 3.1.9.2 are provided as due diligence [44, 45]. Calculations focus on the primary residual change model: a generalized linear model predicting the outcome as a function of the randomized group, with covariate adjustment for baseline (akin to ANCOVA). Assuming two-sided alpha = 0.05, a sample size *N* = 40 provides 80% power to detect Cohen’s *f* = 0.45.

### Experimental procedure and blinding

Anodal tDCS will be applied to the left dorsolateral prefrontal cortex, while cathodal electrode will be positioned on the right dorsolateral prefrontal cortex according to the Omni-Lateral-Electrode-System (OLE-System) [46]. Following a brief training, and under televideo supervision by project staff, caregivers will set up and administer tDCS for participants with AD at home. tDCS will be applied for 30 min at an intensity of 2 mA, with 30 s ramping up and down.

The same procedure will be used for sham stimulation, but in this case, an electric current will be applied at the beginning and the end of 30 s to mimic the perception of active tDCS. All patients, caregivers, and clinicians will be blinded to the type of stimulation delivered. As mentioned, all sessions will be remotely supervised by trained research staff, and sessions will be delivered over five consecutive days (Monday to Friday) for 6 weeks.

The tDCS device will be a 1 × 1 mini-CT stimulator with 5 cm × 7 cm saline pre-saturated sponge electrodes from Soterix Medical. The 1 × 1 mini-CT stimulator contains a built-in code system for blinding in clinical trials. At the beginning of every session, a 6-digit code will be entered into the device to select either sham or active stimulation. Neither participants, caregivers, nor project staff supervisors know what the code means, guaranteeing the blinding process. Caregivers will receive one in-person training session at the day they receive the tDCS device. Project staff will instruct caregivers on how to correctly place the electrodes and how to operate the tDCS device (i.e., turning the device on and off, recharging the batteries, inserting the 6-digit codes, and proper storage of the device).

Participants will be assessed at baseline, during treatment weeks (week 2, week 4, and week 6), and 6 weeks post-treatment (Table 2). At baseline, sociodemographic (age, gender, race/ethnicity) and clinical (medical comorbidities, names, and doses of medications) will be collected.

**Table 2:** Timetable for collection of data

Assessment	Baseline	Week 2	Week 4	Week 6	Week 12
Sociodemographic and clinical information	X				
AES	X	X	X	X	X
bDAS	X	X	X	X	X
Cornell Scale	X			X	X
MMSE	X			X	X
NPI-Q	X	X	X	X	X
tDCS experience questionnaire		X	X	X	X
Side effects questionnaire		X	X	X	X

*Abbreviations: AES* Apathy Evaluation Scale, *bDAS* Brief Dimensional Apathy Scale, *MMSE* Mini-Mental State Exam, *NPI-Q* Neuropsychiatric Inventory, *tDCS* transcranial direct current stimulation

We will use bi-hemispheric stimulation (anode left/cathode right prefrontal cortex) based on prior research showing bilateral frontal circuits are implicated in apathy, and bilateral stimulation may have wider effects on brain networks [19, 47, 48]. Stimulation will last 30 min, consistent with previous studies of older adults [18, 47, 49]. Setting at 6 weeks, the length of our tDCS protocol was based on the duration of previous clinical trials for apathy in AD [12, 13] and the evidence that shorter tDCS was not effective [29].

Home-based tDCS protocols will follow the protocol used in our previous studies [30, 31]. Caregivers will be trained during an in-person baseline visit, and all tDCS sessions will be remotely supervised via secure videoconferencing software by trained research staff for the entire duration of each session to ensure the use of proper technique and to monitor any potential adverse events.

### Primary outcome measures

The primary outcomes will be feasibility, acceptability, and safety of home-based tDCS to AD patients with apathy (Table 3).

**Table 3:** Study outcomes

**Primary outcome measures**
* Feasibility*: Recruitment rate (per month), randomization success, blind success, retention/attrition rates
* Acceptability*: Likert scale (from 0 [strongly disagree] to 10 [strongly agree]) composed of ten affirmatives regarding the use of home-based tDCS. For example, question 1: “It was easy to prepare the device and accessories,” question 7: “I felt confident using the device.” Overall acceptability across groups will be evaluated by descriptive measures of satisfaction ratings [31]
* Safety*: Side effects questionnaire that include itching, burning, headache, fatigue, and dizziness [31]
**Secondary outcome measures (tools)**
-Apathy (AES and bDAS) [39, 40]
-Neuropsychiatric symptoms (NPI-Q) [35, 36]
-Depressive symptoms (Cornell Scale for Depression in Dementia) [38]
-Cognition (MMSE) [33]

All outcome measures are continuous

*Abbreviations: AES* Apathy Evaluation Scale, *bDAS* Brief Dimensional Apathy Scale, *MMSE* Mini-Mental State Exam, *NPI-Q* Neuropsychiatric Inventory, *tDCS* transcranial direct current stimulation

#### Feasibility

Feasibility measures will include recruitment rate (per month), randomization success, blind success, and retention/attrition rates. Feasibility will be considered supported if the current trial demonstrates (a) a recruitment rate of 1–2 participants/month and (b) a retention rate of 80% or higher.

#### Acceptability

Acceptability will be evaluated using the method used in prior tDCS studies by Ahn et al. [31]. Caregivers will be asked to apply a Likert scale (from 0 [strongly disagree] to 10 [strongly agree]) to answer ten affirmatives regarding the use of home-based tDCS. For example, question 1: “It was easy to prepare the device and accessories,” question 7: “I felt confident using the device.” Overall acceptability will be evaluated by descriptive measures of satisfaction ratings.

#### Safety

Safety will be assessed with a questionnaire about side effects that include itching, burning, headache, fatigue, and dizziness [31].

### Secondary outcome measures

In this pilot trial, secondary outcomes focus on the efficacy of tDCS for AD-related symptoms (clinical outcomes), namely apathy (Table 3). Therefore, the central secondary clinical outcome measure will be the Apathy Evaluation Scale (AES) score [39] and the Brief Dimensional Apathy Scale (bDAS) [40]. Apathy will be assessed at baseline, during treatment (weeks 2, 4, and 6), and 6 weeks post-treatment.

Other secondary clinical outcome measures will include (1) total scores on the NPI-Q which evaluates 12 discrete NPS with scores ranging from 0 to 144 [35, 36]; (2) depressive symptoms as assessed by the Cornell Scale for Depression in Dementia [38]; and (3) cognition as evaluated by the MMSE, which includes memory, language, praxis, and orientation tasks, yielding a global cognition score ranging 0 to 30, with higher scores indicating better performance [33].

### Data analysis

For the primary outcomes feasibility, acceptability, and safety, GLM will separately evaluate each of three outcomes as a function of the treatment group: (1) consent, (2) completion rate, and (3) participant satisfaction. Regarding secondary outcomes, GLM will also separately evaluate apathy, depression, NPS, and cognition. Specifically for apathy, GLM will evaluate residual change at the end of treatment as a function of the treatment group, controlling for symptom severity at the beginning of treatment (i.e., Apathy_Endpoint_ = Group + Apathy_Baseline_). Follow-up analyses will evaluate group differences at all other measured time points (post-baseline). This residual change approach is essentially a GLM-based analog to ANCOVA that permits non-normally distributed outcome distributions. Additional follow-up longitudinal analyses will evaluate the functional form of change across all time points between groups via GLMM (i.e., Apathy = Time + Group + Time × Group) with a random effect structure (e.g., random intercept; random slope; random intercept and slope) determined by fit indices (e.g., Akaike information criteria). Potential nonlinear effects will be evaluated via the inclusion of polynomial or spline terms. A similar strategy will be used for each other outcome secondary outcome (in separate models) by modeling scores at the end of treatment as a function of treatment group, controlling for baseline, and GLMM will evaluate the longitudinal trajectory of each outcome across and between groups. Following recent recommendations in the clinical trial literature analyses, we will proceed using parallel frequentist and Bayesian statistical inference [50, 51]. The Bayesian inferential paradigm can provide probabilistic estimates of effects irrespective of sample size.

### Data safety monitoring

All research data will be de-identified. All participants will be given a unique code that will be linked to their personal information accessible only by the researcher team. Given the low-risk nature of the current study, no independent Data Safety Monitoring Board will be established. The members of the research team will actively monitor the fidelity to protocol through regular meetings and peer observation.

## Discussion

Given the growing numbers of older adults with neurodegenerative diseases and the related pressure on health systems, there is an urgent need to develop more effective therapeutic strategies that minimize or halt the progression of AD and to alleviate the related cognitive and behavioral symptoms affecting patients and their families such as AD-related apathy [1].

There is growing literature on the therapeutic role of tDCS in AD, especially focusing on cognitive functioning. Recent systematic reviews and meta-analyses of these studies have shown that tDCS has the potential to improve cognition, mainly in the early stages of the disease, but stimulation parameters (multiple sites; single vs. repeated; lower vs. higher current) were very different among studies, preventing definite conclusions [22–26]. Of relevance, Im et al. investigated changes in cognitive performance, as assessed by the MMSE and other specific neuropsychological tests, after home-based 2 mA tDCS with anodal on the left dorsolateral prefrontal cortex and cathodal on the right dorsolateral prefrontal cortex for 30 min daily for 6 months in patients with early AD [47]. Besides showing the initial feasibility of home-based tDCS, these researchers found that daily tDCS improve or stabilize cognitive decline in patients with AD. Importantly, this clinical effect was associated with changes in the regional cerebral metabolic rate for glucose in the left temporal lobe as assessed by 18F-fluoro-2-deoxyglucose positron emission tomography [47]. More recently, Gangemi et al. also reported that short- (10 days) and long-term (10 days per month for 8 months) 2 mA anodal tDCS for 20 min daily applied on the left prefrontal cortex resulted in slower progression of cognitive decline and neurophysiological changes compared to sham tDCS [52]. Altogether these studies suggest that tDCS is a promising tool for cognitive stabilization in AD.

To date, only one study investigated the effect of tDCS on NPS in AD [53]. Suemoto et al. studied 40 patients with AD who were randomized to receive either anodal tDCS (2 mA constant current for 20 min) or sham-tDCS over the left dorsolateral prefrontal cortex (DLPFC) for six sessions during 2 weeks [29]. While tDCS was safe in this population, there was no evidence of the efficacy of tDCS on apathy nor on other NPS assessed. The observed lack of efficacy was attributed to several factors, including the low number of sessions and the short period of intervention. One important aspect of this study was the challenge to engage subjects in the trial mainly because of issues related to transportation to the medical center for tDCS application [29]. The current study will overcome these potentially limiting factors, offering more sessions for a longer period of time, not requesting the patients to attend any clinic. Furthermore, remote supervision of active tDCS or sham tDCS daily sessions by trained research staff will ensure correct use of the device and increase compliance and likelihood of study completion.

As home-based tDCS has proven feasible in older adults [30, 31], our hypotheses are that participants with AD will tolerate tDCS without significant adverse effects and that active tDCS group will demonstrate lower apathy scores alongside lower scores on the NPI-Q and Cornell Scale for Depression at the end of treatment relative to sham tDCS. Given that research participants will perform MMSE multiple times, it is possible that cognitive performance will improve as a result of practice effect in both sham and active tDCS.

## Conclusion

Our study will address a frequent and sometimes overlooked clinical problem, i.e., NPS with focus on apathy, in patients with AD. Our findings can advance the field of non-pharmacological strategies for NPS, also presenting a great potential for clinical translation. Our expectation is that home-based intervention with real-time monitoring through a secure conferencing platform might be regarded a new modality for improving symptom management in AD.

## Data Availability

Not applicable.
